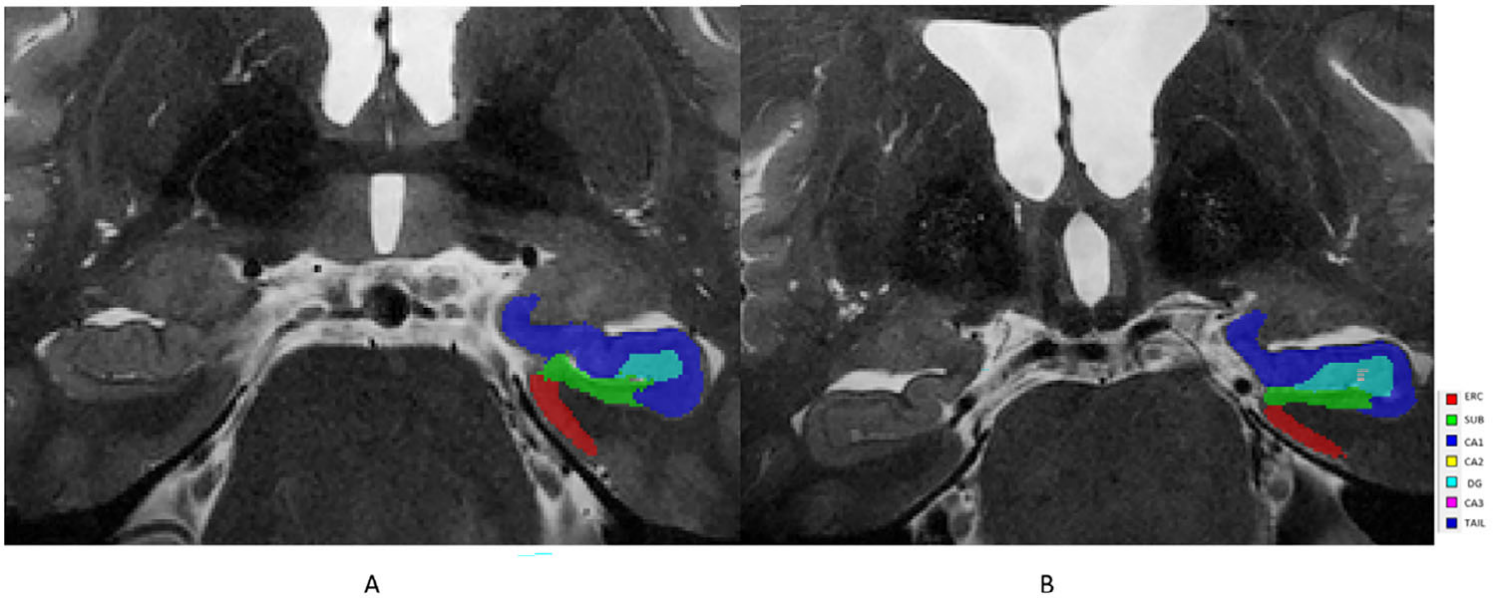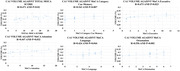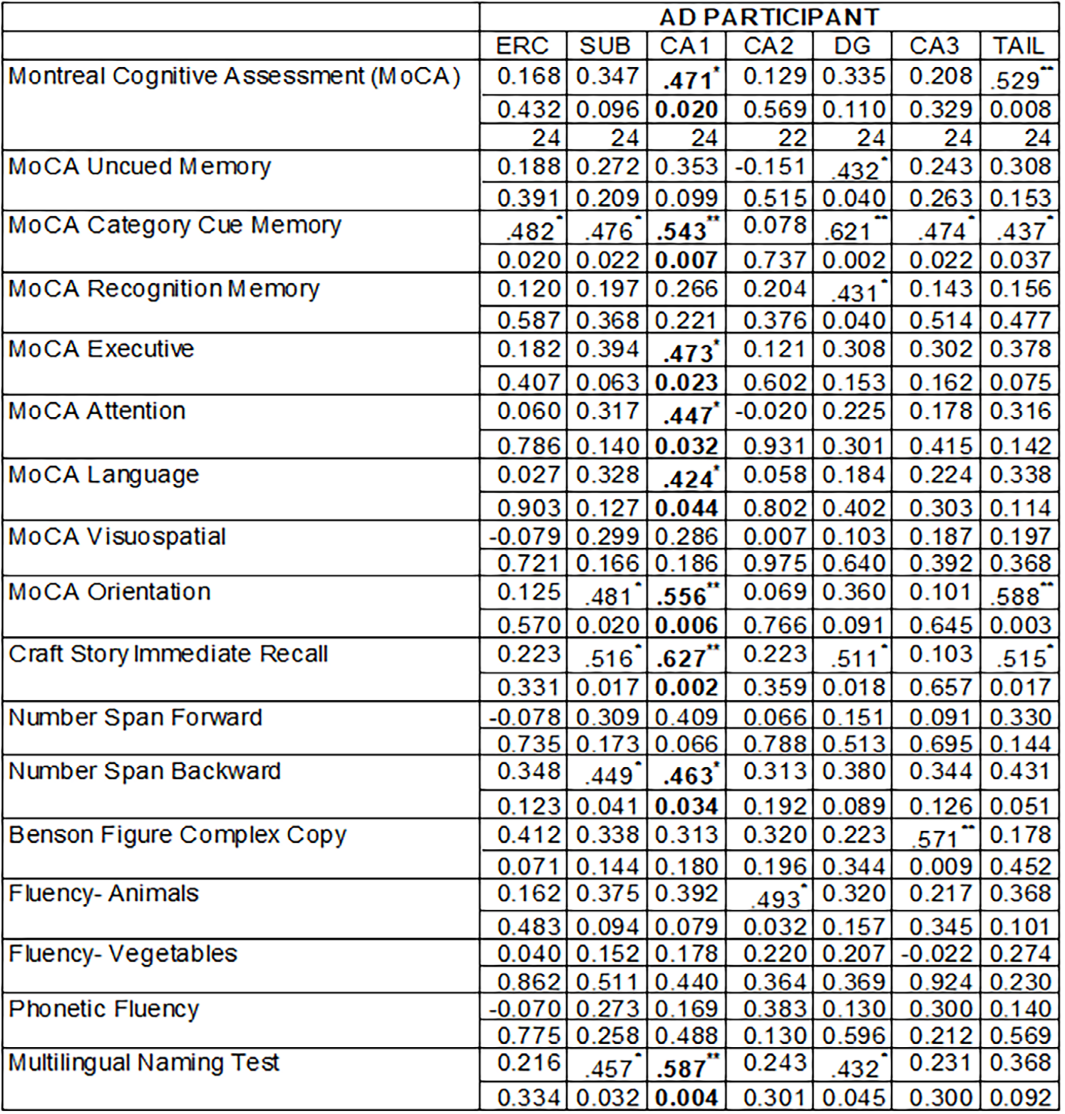# Correlation Between Cognitive Impairment in Early‐Stage Amyloid‐Positive Alzheimer's Disease and CA1 Hippocampal Subfield Volume Loss Using 7T MRI

**DOI:** 10.1002/alz.088815

**Published:** 2025-01-09

**Authors:** Oluwatobi F Adeyemi, Olivier Mougin, Gowland Penny, Richard Bowtell, Elizabeta Mukaetova‐Ladinska, Akram A. Hosseini, Jagrit Shah

**Affiliations:** ^1^ Sir Peter Mansfield Imaging Centre, University of Nottingham, Nottingham UK; ^2^ University of Abuja, Abuja Nigeria; ^3^ School of Psychology, University of Leicester, Leicester, Leicestershire UK; ^4^ Nottingham University Hospitals NHS Trust, Queens Medical Center, Nottingham UK; ^5^ Nottingham University Hospitals NHS Trust, Nottignham UK; ^6^ Nottingham University Hospital Trust, Nottingham, Nottingham UK

## Abstract

**Background:**

Early diagnosis is crucial in Alzheimer's disease (AD) for optimal treatment outcomes. Neuropsychometric assessments, particularly using the Uniform Data Set Neuropsychological Battery (UDSNB3.0) [1], provide insights into cognitive domains in early stages of Alzheimer’s disease before significant hippocampal atrophy occurs. This study leverages 7T MRI, which offers superior spatial resolution, to investigate these correlates.

**Method:**

Forty‐seven participants were recruited (24 AD patients and 23 matched healthy controls [HC]; age range: 40 ‐ 76 years). Participants selected for the study met the ATN criteria [2] for symptomatic Alzheimer's disease with CSF‐Amyloid positivity. Participants with significant hippocampal atrophy (i.e. MTA score of >2 on clinical MRI at 1.5/3T [3] were excluded. Imaging was carried out on a Philips Achieva 7T scanner equipped with a Nova Medical single‐channel transmit and 32‐channel receive (1Tx32Rx) head coil. The acquired images included PSIR sequences (TE/TR=3.1/6.9ms; FA=60°) with isotropic 0.55 mm resolution, as well as T2‐weighted FSE sequences (TE/TR=119/5900ms; FA=90°) with a resolution of 0.38x0.39x1.50 mm³.

**Result:**

The reduction in the volume of CA1 hippocampal subfield in patients with AD significantly correlated with multiple cognitive domains, highlighting its central role in various aspects of cognitive function (see Table 1 and Figure 2). The change in volume was not significant for the whole brain showing that the hippocampus was changing particularly and not simply as part of general brain atrophy.

A significant linear correlation was evident between the volume of CA1 and the total MoCA score (R^2^ = 0.47; p=0.02), indicating a particularly strong correlation with the memory category with p<0.05. There were also significant correlations between CA1 and specific cognitive subdomains including executive function, attention, language, Orientation, Craft Story Immediate, Number Span Backward.

**Conclusion:**

The observed result, indicating a robust correlation between the CA1 hippocampal subfield volume and various cognitive measures, in the early stages of Alzheimer's disease, provides valuable insights into the intricate relationship between neuroanatomy and cognitive function.